# Expression of VEGFA‐regulating miRNAs and mortality in wet AMD

**DOI:** 10.1111/jcmm.14731

**Published:** 2019-10-21

**Authors:** Janusz Blasiak, Cezary Watala, Raimo Tuuminen, Niko Kivinen, Ali Koskela, Hannele Uusitalo‐Järvinen, Anja Tuulonen, Mateusz Winiarczyk, Jerzy Mackiewicz, Szymon Zmorzyński, Agata Filip, Kai Kaarniranta

**Affiliations:** ^1^ Department of Molecular Genetics Faculty of Biology and Environmental Protection University of Lodz Lodz Poland; ^2^ Department of Haemostatic Disorders Medical University Lodz Poland; ^3^ Helsinki Retina Research Group University of Helsinki Helsinki Finland; ^4^ Department of Ophthalmology Kymenlaakso Central Hospital Kotka Finland; ^5^ Department of Ophthalmology University of Eastern Finland Kuopio Finland; ^6^ Department of Ophthalmology Tampere University Hospital Tampere Finland; ^7^ Department of Vitreoretinal Surgery Medical University of Lublin Lublin Poland; ^8^ Department of Cancer Genetics Medical University of Lublin Lublin Poland; ^9^ Department of Ophthalmology Kuopio University Hospital Kuopio Finland

**Keywords:** age‐related macular degeneration, AMD, anti‐VEGFA injection, miRNA, mortality, wet AMD therapy

## Abstract

MicroRNAs (miRNAs) regulate gene expression; many of them act in the retinal pigment epithelium (RPE), and RPE degeneration is known to be a critical factor in age‐related macular degeneration (AMD). Repeated injections with anti‐VEGFA (vascular endothelial growth factor A) are the only effective therapy in wet AMD. We investigated the correlation between the expression of 18 miRNAs involved in the regulation of the *VEGFA* gene in serum of 76 wet AMD patients and 70 controls. Efficacy of anti‐VEGFA treatment was evaluated by counting the number of injections delivered up to 12 years. In addition, we compared the relative numbers of deaths in patient with AMD and control groups. We observed a decreased expression of miR‐34‐5p, miR‐126‐3p, miR‐145‐5p and miR‐205‐5p in wet AMD patients as compared with controls. These miRNAs are involved in the regulation of angiogenesis, cytoprotection and protein clearance. No miRNA was significantly correlated with the treatment outcome. Wet AMD patients had greater mortality than controls, and their survival was inversely associated with the number of anti‐VEGFA injections per year. No association was observed between miRNA expression and mortality. Our study emphasizes the need to clarify the role of miRNA regulation in AMD pathogenesis.

## 1 INTRODUCTION

Age‐related macular degeneration (AMD) is a complex eye disease; it is the leading cause of legal blindness in the elderly in the developed countries. AMD occurs in either dry or wet (neovascular) forms. Wet AMD is characterized by the sprouting of new vessels from choriocapillaris through the Bruch's membrane into the sub‐retinal space or the retina layers. Vascular endothelial growth factor A (VEGFA) and its receptor are crucial regulators of choroidal neovascularization (CNV) in wet AMD.^1^ The vessels produced in CNV are fragile, and their contents tend to leak into the retina layers, promoting fibrogliosis which results in the formation of a disciform scar and severe loss of vision if not properly treated.

Targeting VEGFA by anti‐VEGFA agents significantly improved the treatment of wet AMD outcome and, in fact, led to its removal from the list of incurable diseases.^2^ Bevacizumab (Avastin^®^), ranibizumab (Lucentis^®^) and aflibercept (Eylea^®^) are used to prevent CNV activity. However, not all patients display a positive response to these drugs, and some of them are unresponsive to therapy.^3^ Moreover, the relatively short half‐life of anti‐VEGFA drugs means that they need to be administered on a monthly basis, with the treatment often lasting for the patient's life‐time. Therefore, new modes of treatments of wet AMD with the currently used and new drugs are being investigated to find compounds with greater efficacy and better safety than the present anti‐VEGFA–based therapies.

The goal of anti‐VEGFA therapy in wet AMD is to decelerate the worsening of visual acuity and to prevent the loss of vision. Although the use of VEGFA inhibitors revolutionized wet AMD therapy, several issues still need to be resolved in the treatment of this disease. First, VEGFA is not the only protein involved in neovascularization. Second, neovascularization is not the only unwanted process ongoing in affected eyes. Third, individual susceptibility to therapy is influenced by genotype, epigenotype and environmental factors, all of which determine the expression of genes whose products are important for the therapeutic response.

Wet AMD is frequently associated with other systemic conditions, primarily vascular complications that can be linked with increased mortality.^4^, ^5^, ^6^, ^7^ Therefore, although the question of whether wet AMD may be an independent risk factor for death is complex, it should be addressed as advanced age is the main factor in the pathogenesis of AMD. Moreover, some reports suggest that therapy with intravitreous anti‐VEGFA injections can influence the mortality of wet AMD patients undergoing this kind of therapy.^8^, ^9^, ^10^

Several genetic factors, mainly related to the complement system, have been identified as playing either a documented or a putative role in the pathogenesis of AMD, but the role of epigenetic control in AMD is much less clear.^11^, ^12^, ^13^ As epigenetic microRNAs (miRNAs) are an important element in the regulation of gene expression, a panel of miRNA species involved in the progression of wet AMD or the conversion of dry AMD into wet AMD should be clarified in order to better understand AMD pathogenesis and to personalize wet AMD therapy. miRNAs now have an emerging role in the regulation of the expression of eukaryotic genes, and they are known to be involved in the pathogenesis of many human diseases.^14^ Their main function is to trigger the RNA interference (RNA_i_) pathway, either to degrade mRNA produced by the target gene or to repress its translation. However, several other functions have been attributed to miRNAs, including transcriptional gene activation.^15^ Moreover, a single miRNA can be involved in the control of multiple genes belonging to a single molecular pathway or multiple pathways. A recent study has identified as many as 416 miRNAs which are expressed in RPE and choroid.^16^ Targeting these miRNAs in order to modulate their expression seems to be a promising strategy in AMD treatment as supported by experiments conducted in animal models of AMD.^17^

In the present work, we investigated the expression of *VEGFA* gene‐regulating miRNAs in the serum of wet AMD patients. In addition, we analysed mortality in the wet AMD patients and control groups without AMD.

## 2 MATERIALS AND METHODS

### 2.1 Patients

A total of 76 patients with wet AMD and 70 controls were enrolled in this study. The mean age of the patients was 79.5 years (range 74.5‐84.5), whereas the mean age of controls was 73.8 years (68.6‐78.7). The patient group contained 18 males and 58 females; the numbers in the control group were 37 males and 33 females. Because of the differences in age and sex, all calculations were adjusted for these parameters. The controls were individuals without AMD or any other retinal disease who were undergoing cataract operation and were selected as described earlier.^18^ The criteria for patient selection were based on CNV in optical coherent tomography (OCT) and/or fluorescein angiography (FAG). No patient reported any genetic disease, and diabetes mellitus was an exclusion criterion. All patients with AMD were subjected to an examination in the Department of Ophthalmology of Kuopio University Hospital, involving best‐corrected visual acuity (VA), intraocular pressure, slit lamp, fundus and biomicroscopy examination, fundus photographs (Canon CX‐1 Hybrid Retinal Camera, Canon), FAG (Canon CX‐1) and/or OCT (SPECTRALIS OCT2, Heidelberg Engineering). Real‐world data (RWD) were monitored for up to 12 years. Finnish national guidelines for modified PRN (*pro re nata*) were applied in the follow‐up and treatments of wet AMD patients.^19^, ^20^, ^21^, ^22^

Ethics Committee of the Kuopio University Hospital has approved the study, and the tenets of the Declaration of Helsinki are followed. All participants have been asked to sign an informed consent form.

### 2.2 Cataract surgery and wet AMD registries

Cataract surgery, wet AMD and anti‐VEGFA registries were gathered at the Department of Ophthalmology, Kymenlaakso Central Hospital, Kotka, Finland. The registries of operations for phacoemulsification cataract surgery (ICD code: CJE20), of patients with wet AMD (ICD code: H35.31) and of intravitreal injections (ICD code: CKD05), all from 03 September 2007, were combined. Patients under 65 years were excluded from the data analysis. Patient age and sex were registered as confounders. The study was approved by the Research Director and Chief Medical Officer of the Kymenlaakso Central Hospital, and the tenets of the Declaration of Helsinki were followed. Each patient gave a written informed consent.

### 2.3 Blood samples, RNA isolation and miRNA expression

Blood samples were taken in the active phase of wet AMD and centrifuged with a serum aliquot of 200 μl being used to isolate RNA with miRCURY Isolation Kit™ (Exiqon). The small RNA fraction was concentrated; the amount of elution buffer was reduced to 20 µL. The robustness of the RNA isolation and the quality of isolated RNA samples were checked using miRCURY microRNA QC PCR Panel (Exiqon). Quantitative real‐time PCR (real‐time qPCR) was performed with miRCURY LNA Universal RT microRNA PCR and MicroRNA LNA PCR primer set (Exiqon). Reverse transcription was carried in a total volume of 10 µL using 10 ng RNA. In the qt‐PCR experiments, cDNA was diluted 80× with nuclease‐free water and an aliquot of 4 µL was used for a 10 µL reaction. Real‐time qPCR was performed using ABI 7500 Fast instrument (Thermo Fisher Scientific). The PCR conditions were as follows: 10 minutes denaturation at 95°C followed by 45 amplification cycles at 95°C 10 seconds, 60°C 1 minute, ramp‐rate of cooling 1.6°C/s. Every sample was assayed in duplicate with expression being calculated according to the 2‐ΔΔCt method.^23^, ^24^ The expression of hsa‐miR‐423‐5p and hsa‐miR‐425‐5p served as a control. miRNAs targeting the VEGFA gene expression were chosen on the basis of the data from the miRTarBase (http://mirtarbase.mbc.nctu.edu.tw) and reference,^25^ and verified using TargetScanHuman database (http://www.targetscan.org). Only those miRNAs were selected which had been experimentally validated with evidence emerging from reliable methods. They are listed in Table 1.

**Table 1:** miRNAs targeting the *VEGFA* (vascular endothelial growth factor A) gene

miRNA	miRBase/miRTarBase entry
hsa‐miR‐15a‐5p	MIMAT0000068/MIRT004275
hsa‐miR‐16‐5p	MIMAT0000069/MIRT003890
hsa‐miR‐17‐5p	MIMAT0000070/MIRT025302
hsa‐miR‐20a‐5p	MIMAT0000075/MIRT004450
hsa‐miR‐20b‐5p	MIMAT0001413/MIRT004451
hsa‐miR‐29b‐3p	MIMAT0000100/MIRT003813
hsa‐miR‐34a‐5p	MIMAT0000255/MIRT004513
hsa‐miR‐93‐5p	MIMAT0000093/MIRT004055
hsa‐miR‐106a‐5p	MIMAT0000103/MIRT004465
hsa‐miR‐106b‐5p	MIMAT0000680/MIRT004466
hsa‐miR‐125a‐5p	MIMAT0000443/MIRT004445
hsa‐miR‐126‐3p	MIMAT0000445/MIRT003428
hsa‐miR‐145‐5p	MIMAT0000437/MIRT006215
hsa‐miR‐195‐5p	MIMAT0000461/MIRT004273
hsa‐miR‐200b‐3p	MIMAT0000318/MIRT006440
hsa‐miR‐205‐5p	MIMAT0000266/MIRT004518
hsa‐miR‐361‐5p	MIMAT0000703/MIRT004447
hsa‐miR‐378a‐3p	MIMAT0000732/MIRT004277

### 2.4 Data analysis

Data of miRNA expression are displayed by the bootstrap‐boosted mean ± standard deviation (SD). The number of injections per year in groups is presented as the bootstrap‐boosted medians and interquartile ranges (lower [LQ, 25%] to upper [UQ, 75%] quartile). The significance of differences in miRNA expression in controls and wet AMD patients was estimated with bootstrap‐boosted unpaired Student's *t* test and further validated by using the Benjamini‐Hochberg correction (False Discovery rate (FDR) = 0.25). In the multivariate comparison analysis of miRNA expressions, the bootstrap‐boosted Hotelling T test was applied. Mortality data were presented as odds ratios (OR) and the 95% confidence interval (95% CI) range. The estimates were calculated with the bootstrap‐boosted logistic regression (1000 iterations); OR was adjusted for age, sex and time of observation; and the goodness of fit in the model was estimated with the Hosmer‐Lemeshow statistics. The comparisons of death ratios and the numbers of injection per year between groups were estimated with the Fisher exact test or the bootstrap‐boosted Mann‐Whitney *U* test, respectively. All the bootstrapped estimates, except for the logistic regression, were performed with 10 000 iterations. For cataract surgery, wet AMD and anti‐VEGFA registries, statistical analysis was performed using IBM SPSS Statistics 25 (SPSS Inc). Kaplan‐Meier curves were generated, and Cox regression was used to estimate hazard ratios (HR) for death between patients with wet AMD and anti‐VEGFA injections and the cohort (Tables S1 and S2). HRs were adjusted for confounders including patient age and sex. *P*‐values .05 or less were considered as statistically significant.

## 3 RESULTS

**Figure 1:**
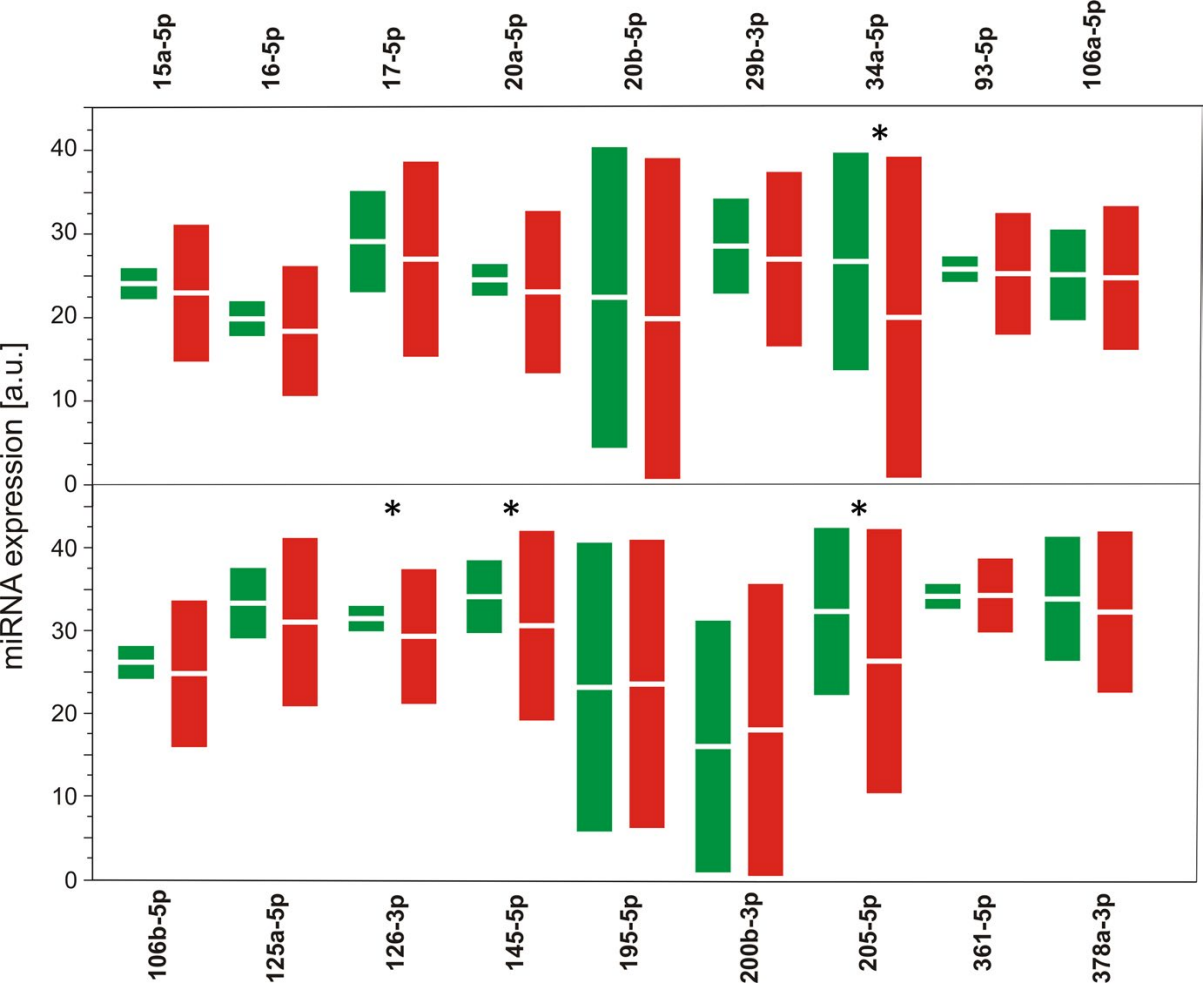
Relative expression of the miRNAs involved in the regulation of the *VEGFA* gene in serum of wet AMD patients (red) as compared with controls (green). Data are shown as bootstrap‐boosted mean ± SD; the bootstrap‐boosted Mann‐Whitney *U* test was used to calculate the Benjamini‐Hochberg‐corrected *P* values, and in the multivariate comparison analysis, the bootstrap‐boosted Hotelling T test was applied. Significance of differences estimated with bootstrap‐boosted unpaired Student's *t* test and further validated with the use of Benjamini‐Hochberg correction (FDR = 0.25); N = 76 for patients with AMD and 70 for controls; *P < .05

Four out of 18 miRNAs regulating the expression of the *VEGFA* gene displayed a lower level of expression in patients with AMD than in controls: miR‐34a‐5p, miR‐126‐3p, miR‐145‐5p and miR‐205‐5p (Figure 1). We observed a significantly elevated mortality ratio in wet AMD patients in comparison with controls (*P* = .021, Table 2).

**Table 2:** Mortality in wet AMD patients and controls^a^

Group	Death ratio	OR	95% CI	*P*
Wet AMD (76)	0.276	4.180	(1.228; 16.683)	.021
Controls (70)	0.085			

a Data presented as the odds ratio and the 95% confidence interval (95% CI) range. The estimates were calculated with the bootstrap‐boosted logistic regression (1000 iterations); OR was adjusted for age, sex and time of observation; and the goodness of fit of the model was estimated with the Hosmer‐Lemeshow statistics (*P* = .450).

**Figure 2:**
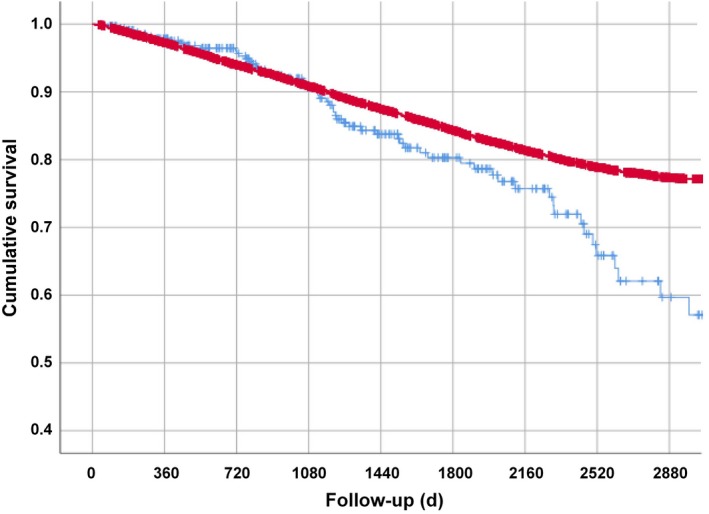
Registry of cataract surgery patients operated after 3 September 2007. Patients with wet AMD (ICD code: H35.31) and intravitreal injections (ICD code: CKD05) (blue) were compared to the cataract cohort (red). Kaplan‐Meier curves were generated, and Cox regression was used to estimate hazard ratios (HR) for death between wet AMD patients with anti‐VEGFA injections and cataract cohort. All patients were 65 years or older. In wet AMD patients, after adjusting for age and sex, HR for death was 2.05; 95% CI 1.59‐2.64; *P* < .001 when compared to cataract surgery patients without wet AMD. N = 330 for wet AMD patients and N = 15 364 for the cataract cohort

The wet AMD patients who died during the treatment had received a higher number (>3 injections/year (*P* = .0013) of anti‐VEGFA injections than those who survived (Table 3). In Cox regression analysis adjusted for age and sex, cataract surgery patients with wet AMD and anti‐VEGFA injections (mean number of 9.8 ± 8.9 anti‐VEGFA injections during the follow‐up) had HR 2.05 for death, 95% CI 1.59‐2.64, when compared to the cataract surgery patients without wet AMD (*P* < .001, Figure 2). No association was observed between mortality and the expression of any miRNA (data not shown).

**Table 3:** Average number of anti‐VEGFA injections per year in wet AMD patients who died during the treatment and those who survived^a^

Eye	Status	Injections/year	*P*
Left	Dead	3.28 (1.90; 4.38)	.0013
	Survived	1.31 (0.09; 3.25)	
Right	Dead	2.65 (1.28; 3.98)	.068
	Survived	1.19 (0.06; 2.53)	

a Data presented as median and interquartile range (lower [LQ, 25%] to upper [UQ, 75%] quartile); n = 54 survivors and n = 20 non‐survivors. Significance estimated with the Mann‐Whitney *U* test. All estimates (median, LQ, UQ and Mann‐Whitney *U* statistics) were bootstrap‐boosted with 10 000 iterations.

## 4 DISCUSSION

MiRNAs are an emerging topic, for example as biomarkers and therapeutic targets of diseases, including AMD. We observed decreased serum levels of miR‐34‐5p, miR‐126‐3p, miR‐145‐5p and miR‐205‐5p in wet AMD patients as compared with non‐AMD controls.

The expression of miR‐34a‐5p may occur in response to oxidative stress, a major factor of AMD pathogenesis.^26^ miR‐34a‐5p negatively regulates angiogenesis, which is essential for the development of wet AMD.^27^, ^28^ The up‐regulation of miR‐34a‐5p was reported to be involved in drusen formation in AMD through the down‐regulation of the triggering receptor expressed in myeloid/microglial cells‐2 (TREM2).^29^ This receptor is involved in the clearance of the aggregated Aβ42 peptide from the extracellular space. One target of miR‐34a‐5p is heat shock protein family A (Hsp70) that functions as a molecular chaperone and exerts a cytoprotective effect by refolding proteins that have been misfolded in the presence of oxidative stress.^30^ Decreased miR‐34a‐5p serum levels in wet AMD may evoke increased cellular stress and microglia activation that lead to the wet AMD phenotype. miR‐126‐3p belongs to the angiomiR family of miRNAs which are involved in regulating angiogenesis. The role of miR‐126 in the promotion of angiogenesis was first correlated with targeting of the *Spred‐1* gene, which encodes for a negative regulator of MAP kinase signalling.^31^ miR‐126 has been identified as a positive regulator of angiogenesis in chronic heart failure and a negative regulator of the inflammatory response in human cardiac microvascular endothelial cells.^32^, ^33^ Zhou and colleagues observed that the function of miR‐126 was strand and cell‐type specific in animal models of ocular angiogenesis as well as in a mouse model of wet AMD.^34^ They reported that miR‐126‐3p repressed *VEGFA* expression in RPE cells in two ways: directly targeting *VEGFA* 3’‐UTR and by a novel mechanism involving the regulation of αB‐crystallin promoter activity. However, overexpression of miR‐126p enhanced laser‐induced choroidal neovascularization. In addition to the VEGFA control, miR‐126‐3p down‐regulation is associated with increased inflammation, epithelial‐mesenchymal transition (EMT), regulatory proteins expression and cellular proliferation.^35^, ^36^, ^37^, ^38^ Therefore, miR‐126‐3p may be an important epigenetic regulator in the development of wet AMD. miR‐145‐5p is known to be a negative regulator of angiogenesis.^39^ Decreased levels miR‐145‐5p levels enhance the secretion of IL‐1β, TNF‐α and IL‐6 during hypoxia.^40^ Therefore, miR‐145‐5p can be considered as a therapeutic target to suppress the inflammatory response and to prevent the apoptosis occurring in hypoxic conditions, a characteristic of wet AMD.^41^, ^42^ miR‐205‐5p was shown to regulate EMT through the PI3K/AKT pathway.^43^, ^44^ Autophagy, a key lysosomal clearance mechanism, has been linked to the PI3K/AKT signalling.^45^ Impaired autophagy is related to increased inflammation, extracellular matrix remodelling and RPE degeneration in AMD, and the EMT is involved in choroidal neovascularization (CNV) in wet AMD.^46^, ^47^ A direct relationship between miR‐205 and *VEGFA* was confirmed in human breast cancer cells, when the suppression of endogenous miR‐205 resulted in an increase in the expression of VEGFA.^48^

There is a complex network of miRNA interactions regulating gene expression; that is, a single miRNA may be involved in the control of up to 200 genes and a single transcript may have recognition sites for many miRNAs.^49^ Variations in miRNA expression levels may be caused by different factors—host gene mutations or polymorphisms, alterations of proteins associated with miRNA biogenesis or the effects of related long non‐coding RNAs (lncRNAs). For example, the expression of VEGFA may be up‐regulated by lncRNA MALAT1, which targets miR‐145, resulting in the promotion of angiogenesis in brain microvascular endothelial cells.^50^ In summary, miR‐34a‐5p, miR‐126‐3p, miR‐145‐5p and miR‐205‐5p may regulate cellular inflammation, modifications to both the extracellular matrix and phenotype as well as altering cellular proliferation all of which may contribute to the clinical signs of wet AMD.

Ertekin et al found that 11 miRNAs (miR‐21, miR‐25‐3p, miR‐140, miR‐146b‐5p, miR‐192, miR‐335, miR‐342, miR‐374a, miR410, miR‐574‐3p and miR‐660‐5p) were down‐regulated and five others (miR‐17‐5p, miR‐20a, miR‐24, miR‐106a and miR‐223) up‐regulated in the plasma of 33 wet AMD patients.^51^ None of these miRNAs were studied in our cohort. The role of genetic background in the regulation of inflammation and neovascularization was supported by the bioinformatics data reported by Strafella et al, showing an association of rs11671784 (miR‐27A, G > A) and rs2910164 (miR‐146A, C > G) single nucleotide polymorphisms in AMD.^52^ The complexity of AMD pathogenesis increases when non‐genetic data are analysed with genetic and epigenetic data.^53^ However, a bioinformatics analysis of epigenetics, pharmacogenetics, comorbidities and genetic counselling in AMD is a promising way to open new perspectives for personalized medicine and help to identify phenotype differences between dry and wet AMD.^53^, ^54^, ^55^, ^56^

Anti‐VEGFA therapy in wet AMD is considered to be safe for patients, even although some side‐effects have been reported.^12^, ^57^ Moreover, Papudesu et al showed that AREDS2 participants with wet AMD in one eye at baseline had a statistically significant increased risk for mortality compared with patients having no or only a few drusen.^58^ Moreover, a visual acuity less than 20/40 was associated with a reduced survival. In our material, 93% of patients had a visual acuity equal or less than 20/40 in the treated eye after follow‐up of one year. Those patients who had received anti‐VEGFA therapy had a greater risk of mortality than non‐treated individuals. In another population‐based study, Dalvin et al concluded that anti‐VEGFA therapy in wet AMD patients was not associated with mortality as compared with non‐treated individuals, including patients with AMD.^59^ Gopinah et al detected a positive correlation between advanced AMD and mortality.^60^ Hanhart et al noted an increased mortality in wet AMD patients after myocardial infarction (MI) treated with bevacizumab as compared to non‐treated individuals.^9^, ^10^ Previously, these authors also observed increased mortality in bevacizumab‐treated wet AMD patients as compared to a control cohort without AMD.^8^ Recent meta‐analyses on mortality of wet AMD patients treated with bevacizumab intravitreal injections have pointed to a less than 2% mortality ratio in the first year of therapy.^61^ A study of over 20 000 participants from the Melbourne Collaborative Cohort Study led to the conclusion that late AMD was linked with an increased mortality.^62^ We detected a relatively high mortality ratio in the wet AMD group, and this did not correlate with alterations in the expression of the investigated miRNAs. We are unaware of any previous studies which have investigated whether anti‐VEGFA therapy is linked with either mortality or miRNAs. Nonetheless, our results should not be interpreted that the anti‐VEGFA therapy is a cause of increased mortality. As AMD is a complex disease associated with both genetic and cardiovascular risk factors, it can be viewed as an expression of the serious poor general health state of these patients that may contribute to their premature death, especially in individuals who require more than the average number of anti‐VEGFA injections. However, we cannot exclude the possibility that anti‐VEGFA therapy has detrimental effects in certain patients.

The correlation between the serum miRNAs profile and wet AMD points to their involvement in the molecular mechanisms underlying AMD pathogenesis, such as inflammation, protein clearance, lipid metabolism or reaction to oxidative stress. In the era of targeted treatment, some circulating miRNAs may become reliable diagnostic, prognostic and predictive factors in AMD. Our study included AMD‐free controls, which raises the question of whether the experimental design was appropriate. Although it could be argued that the best controls would be wet AMD patients with no anti‐VEGFA injections, in the present era no such subpopulation exists. Using data from a subpopulation from the past would introduce a time bias, which could not be reliably controlled. Dry AMD does not seem to be a good control for wet AMD, as most of dry AMD patients never develop wet AMD.^63^

The positive association between mortality and the number of anti‐VEGFA injections in wet AMD patients should be understood as an association between mortality and the severity of the disease and by no means as a direct link between mortality and the dose of an anti‐VEGFA drug. To emphasize this point, please note that our reasoning is based on the following argument. With the use of both simple, bivariate (eg correlation) and multivariate (eg logistic regression) statistical analyses, we have shown that the mortality of the patients and the number of anti‐VEGFA injections were significantly associated. Correlation between the number of anti‐VEGFA injections and patient survival is susceptible to many biases that are beyond these data analysis. Patients with high number of anti‐VEGFA injections tend to represent more aggressive and chronic form of wet AMD when compared to those with low number of anti‐VEGFA injections. On the other hand, anti‐VEGFA treatment is recommended to be discontinued in those patients with recent cardiovascular event or otherwise poor physical condition, when no expectations for ability to function and quality of life effect exist, and in cases of anti‐VEGFA non‐responders or other adverse effects related to the treatment. Furthermore, we have demonstrated the not unexpected significant association between mortality and the patient's age. However, when age was included as a confounding variable and used to standardize (adjust) the association between mortality and number of anti‐VEGFA injections, this association became greatly reduced (although it yet remained statistically significant). In our opinion, this is the most elegant statistical proof of what we have stated in our paper. Specifically, as our analysis was adjusted for the age of the patients with AMD, we may hypothesize that it is unlikely that the observed effect was not purely coincidental but instead related to an advanced age of the patients. It is noteworthy however that although advanced age is the main factor in the pathogenesis of AMD, it is not the only significant factor and not all AMD‐related phenomena can be attributed simply to ageing.

Although our samples are unique as they represent an observation period of up to 12 years, their number is not impressive from the statistical point of view. That is why we applied a bootstrapped estimate with 10 000 iterations to minimize the chance of introducing bias into our analyses. We emphasize that this is a common way to validate conclusions drawn from clinical studies evaluating not very large groups. With such an approach, we attempted to minimize the risk that we would be too eager to reject the null hypothesis when identifying a significant outcome in our inference or association tests. Our study has several limitations, which should be addressed in future investigations. For example, the expression levels of the miRNAs and VEGFA should be evaluated on a year‐by‐year basis in each patient and control individual. The number of patients enrolled in the study as well as the repertoire of miRNAs could be extended, but overall our results support the need for clarifying the role of miRNA regulation in the pathogenesis of AMD.

## CONFLICT OF INTEREST

The authors confirm that there are no conflicts of interest.

## AUTHOR CONTRIBUTION

JB wrote the paper; CW performed statistical analysis; RT performed mortality analysis and wrote the paper; NK gathered clinical data; AK, HU‐J and AT designed experiments and wrote the paper; MW, JM and SZ analysed the data; AF designed experiments and wrote the paper; KK conceived the concept of this work, collected the samples, did clinical examinations, interpreted data, organized financial support and wrote the paper.

## Data Availability

The data that support the findings of this study are available on request from the corresponding author. The data are not publicly available because of privacy and ethical restrictions and are stored at www.kuh.fi.

## References

[1] Shibuya M . Vascular endothelial growth factor (VEGF) and its receptor (VEGFR) signaling in angiogenesis: a crucial target for anti‐ and pro‐angiogenic therapies. Genes Cancer. 2011;2:1097‐1105. 22866201 10.1177/1947601911423031 PMC3411125

[2] Schmidt‐Erfurth U , Chong V , Loewenstein A , et al. Guidelines for the management of neovascular age‐related macular degeneration by the European Society of Retina Specialists (EURETINA). Br J Ophthalmol. 2014;98:1144‐1167. 25136079 10.1136/bjophthalmol-2014-305702 PMC4145443

[3] Mantel I , Gillies MC , Souied EH . Switching between ranibizumab and aflibercept for the treatment of neovascular age‐related macular degeneration (nAMD). Surv Ophthalmol. 2018. 10.1016/j.survophthal.2018.02.004 29476754

[4] Buch H , Vinding T , la Cour M , Jensen GB , Prause JU , Nielsen NV . Age‐related maculopathy: a risk indicator for poorer survival in women: the Copenhagen city eye study. Ophthalmology. 2005;112:305‐312. 15691568 10.1016/j.ophtha.2004.08.025

[5] Fisher DE , Jonasson F , Eiriksdottir G , et al. Age‐Related Macular Degeneration and Mortality in Community‐Dwelling Elders: The Age. Gene/environment susceptibility Reykjavik study. Ophthalmology. 2015;122:382‐390. 25264026 10.1016/j.ophtha.2014.08.006 PMC4306612

[6] Pedula KL , Coleman AL , Yu F , et al. the Study of Osteoporotic Fractures Research G . Age‐related macular degeneration and mortality in older women: the study of osteoporotic fractures. J Am Geriatr Soc. 2015;63:910‐917. 25941039 10.1111/jgs.13405 PMC4439266

[7] Tan J , Wang JJ , Liew G , Rochtchina E , Mitchell P . Age‐related macular degeneration and mortality from cardiovascular disease or stroke. Br J Ophthalmol. 2008;92:509. 18310310 10.1136/bjo.2007.131706

[8] Hanhart J , Comaneshter DS , Freier Dror Y , Vinker S . Mortality in patients treated with intravitreal bevacizumab for age‐related macular degeneration. BMC Ophthalmol. 2017;17:189. 29017506 10.1186/s12886-017-0586-0 PMC5635499

[9] Hanhart J , Comaneshter DS , Freier‐Dror Y , Vinker S . Mortality associated with bevacizumab intravitreal injections in age‐related macular degeneration patients after acute myocardial infarct: a retrospective population‐based survival analysis. Graefes Arch Clin Exp Ophthalmol. 2018;256:651‐663. 29429131 10.1007/s00417-018-3917-9

[10] Hanhart J , Comaneshter DS , Vinker S . Mortality after a cerebrovascular event in age‐related macular degeneration patients treated with bevacizumab ocular injections. Acta Ophthalmol. 2018;96:e732‐e739. 29660843 10.1111/aos.13731

[11] Blasiak J , Petrovski G , Vereb Z , Facsko A , Kaarniranta K . Oxidative stress, hypoxia, and autophagy in the neovascular processes of age‐related macular degeneration. Biomed Res Int. 2014;2014:768026. 24707498 10.1155/2014/768026 PMC3950832

[12] Gemenetzi M , Lotery AJ , Patel PJ . Risk of geographic atrophy in age‐related macular degeneration patients treated with intravitreal anti‐VEGF agents. Eye (Lond). 2017;31:1‐9. 27716750 10.1038/eye.2016.208 PMC5233933

[13] Hjelmeland LM . Dark matters in AMD genetics: epigenetics and stochasticity. Invest Ophthalmol Vis Sci. 2011;52:1622‐1631. 21429863 10.1167/iovs.10-6765 PMC3101660

[14] Bartel DP . Metazoan microRNAs. Cell. 2018;173:20‐51. 29570994 10.1016/j.cell.2018.03.006 PMC6091663

[15] Xiao M , Li J , Li W , et al. MicroRNAs activate gene transcription epigenetically as an enhancer trigger. RNA Biol. 2017;14:1326‐1334. 26853707 10.1080/15476286.2015.1112487 PMC5711461

[16] Karali M , Persico M , Mutarelli M , et al. High‐resolution analysis of the human retina miRNome reveals isomiR variations and novel microRNAs. Nucleic Acids Res. 2016;44:1525‐1540. 26819412 10.1093/nar/gkw039 PMC4770244

[17] Berber P , Grassmann F , Kiel C , Weber BH . An Eye on Age‐Related Macular Degeneration: The Role of MicroRNAs in Disease Pathology. Mol Diagn Ther. 2017;21:31‐43. 27658786 10.1007/s40291-016-0234-z PMC5250647

[18] Kaarniranta K , Paananen J , Nevalainen T , et al. Adiponectin receptor 1 gene (ADIPOR1) variant is associated with advanced age‐related macular degeneration in Finnish population. Neurosci Lett. 2012;513:233‐237. 22387454 10.1016/j.neulet.2012.02.050

[19] Kataja M , Hujanen P , Huhtala H , Kaarniranta K , Tuulonen A , Uusitalo‐Jarvinen H . Outcome of anti‐vascular endothelial growth factor therapy for neovascular age‐related macular degeneration in real‐life setting. Br J Ophthalmol. 2018;102:959‐965. 29074495 10.1136/bjophthalmol-2017-311055 PMC6047152

[20] Tuuminen R , Sipila R , Komulainen J , Saarela V , Kaarniranta K , Tuulonen A . The first ophthalmic Choosing Wisely recommendations in Finland for glaucoma and wet age‐related macular degeneration. Acta Ophthalmol. 2019. 10.1111/aos.14031 30659781

[21] Tuuminen R , Tuulonen A , Kaarniranta K . The Finnish national guideline for diagnosis, treatment and follow‐up of patients with wet age‐related macular degeneration. Acta Ophthalmol. 2017;95:649‐650. 29110437 10.1111/aos.13341

[22] Tuuminen R , Uusitalo‐Jarvinen H , Aaltonen V , et al. The Finnish national guideline for diagnosis, treatment and follow‐up of patients with wet age‐related macular degeneration. Acta Ophthalmol. 2017;95:1‐9. 10.1111/aos.13501 28686003

[23] Livak KJ , Schmittgen TD . Analysis of relative gene expression data using real‐time quantitative PCR and the 2(‐Delta Delta C(T)) Method. Methods. 2001;25:402‐408. 11846609 10.1006/meth.2001.1262

[24] Schmittgen TD , Livak KJ . Analyzing real‐time PCR data by the comparative C(T) method. Nat Protoc. 2008;3:1101‐1108. 18546601 10.1038/nprot.2008.73

[25] Arcondéguy T , Lacazette E , Millevoi S , Prats H , Touriol C . VEGF‐A mRNA processing, stability and translation: a paradigm for intricate regulation of gene expression at the post‐transcriptional level. Nucleic Acids Res. 2013;41:7997‐8010. 23851566 10.1093/nar/gkt539 PMC3783158

[26] Baker JR , Vuppusetty C , Colley T , et al. Oxidative stress dependent microRNA‐34a activation via PI3Kα reduces the expression of sirtuin‐1 and sirtuin‐6 in epithelial cells. Sci Rep. 2016;6:35871‐35871. 27767101 10.1038/srep35871 PMC5073335

[27] Roy A , Zhang M , Saad Y , Kolattukudy PE . Antidicer RNAse activity of monocyte chemotactic protein‐induced protein‐1 is critical for inducing angiogenesis. Am J Physiol Cell Physiol. 2013;305:C1021‐1032. 24048733 10.1152/ajpcell.00203.2013 PMC3840202

[28] Zhao T , Li J , Chen AF . MicroRNA‐34a induces endothelial progenitor cell senescence and impedes its angiogenesis via suppressing silent information regulator 1. Am J Physiol Endocrinol Metab. 2010;299:E110‐116. 20424141 10.1152/ajpendo.00192.2010 PMC2904051

[29] Bhattacharjee S , Zhao Y , Dua P , Rogaev EI , Lukiw WJ . microRNA‐34a‐mediated down‐regulation of the microglial‐enriched triggering receptor and phagocytosis‐sensor TREM2 in age‐related macular degeneration. PLoS ONE. 2016;11:e0150211. 26949937 10.1371/journal.pone.0150211 PMC4780721

[30] Ryhänen T , Hyttinen JM , Kopitz J , et al. Crosstalk between Hsp70 molecular chaperone, lysosomes and proteasomes in autophagy‐mediated proteolysis in human retinal pigment epithelial cells. J Cell Mol Med. 2009;13:3616‐3631. 19017362 10.1111/j.1582-4934.2008.00577.x PMC4516511

[31] Wang S , Aurora AB , Johnson BA , et al. The endothelial‐specific microRNA miR‐126 governs vascular integrity and angiogenesis. Dev Cell. 2008;15:261‐271. 18694565 10.1016/j.devcel.2008.07.002 PMC2685763

[32] Jakob P , Doerries C , Briand S , et al. Loss of angiomiR‐126 and 130a in angiogenic early outgrowth cells from patients with chronic heart failure: role for impaired in vivo neovascularization and cardiac repair capacity. Circulation. 2012;126:2962‐2975. 23136161 10.1161/CIRCULATIONAHA.112.093906

[33] Yang HH , Chen Y , Gao CY , Cui ZT , Yao JM . Protective effects of microrna‐126 on human cardiac microvascular endothelial cells against hypoxia/reoxygenation‐induced injury and inflammatory response by activating PI3K/Akt/eNOS signaling pathway. Cell Physiol Biochem. 2017;42:506‐518. 28578351 10.1159/000477597

[34] Zhou Q , Anderson C , Hanus J , et al. Strand and cell type specific function of microRNA‐126 in angiogenesis. Mol Ther. 2016;24:1823‐1835. 27203443 10.1038/mt.2016.108 PMC5112035

[35] Fish JE , Santoro MM , Morton SU , et al. miR‐126 regulates angiogenic signaling and vascular integrity. Dev Cell. 2008;15:272‐284. 18694566 10.1016/j.devcel.2008.07.008 PMC2604134

[36] Caporali S , Amaro A , Levati L , et al. miR‐126‐3p down‐regulation contributes to dabrafenib acquired resistance in melanoma by up‐regulating ADAM9 and VEGF‐A. J Exp Clin Cancer Res. 2019;38:272. 31227006 10.1186/s13046-019-1238-4 PMC6588909

[37] Xi T , Jin F , Zhu Y , et al. MicroRNA-126-3p attenuates blood-brain barrier disruption, cerebral edema and neuronal injury following intracerebral hemorrhage by regulating PIK3R2 and Akt. Biochem Biophys Res Commun. 2017;494:144–151. 29042193 10.1016/j.bbrc.2017.10.064

[38] Liu R , Zhang YS , Zhang S , et al. MiR‐126‐3p suppresses the growth, migration and invasion of NSCLC via targeting CCR1. Eur Rev Med Pharmacol Sci. 2019;23:679‐689. 30720175 10.26355/eurrev_201901_16881

[39] Climent M , Quintavalle M , Miragoli M , Chen J , Condorelli G , Elia L . TGFbeta triggers miR‐143/145 transfer from smooth muscle cells to endothelial cells, thereby modulating vessel stabilization. Circ Res. 2015;116:1753‐1764. 25801897 10.1161/CIRCRESAHA.116.305178

[40] Yuan M , Zhang L , You F , et al. MiR‐145‐5p regulates hypoxia‐induced inflammatory response and apoptosis in cardiomyocytes by targeting CD40. Mol Cell Biochem. 2017;431:123‐131. 28281187 10.1007/s11010-017-2982-4

[41] Blasiak J , Petrovski G , Veréb Z , Facskó A , Kaarniranta K . Oxidative stress, hypoxia, and autophagy in the neovascular processes of age‐related macular degeneration. Biomed Res Int. 2014;2014:768026. 24707498 10.1155/2014/768026 PMC3950832

[42] Stefánsson E , Olafsdottir OB , Eliasdottir TS , et al. Retinal oximetry: metabolic imaging for diseases of the retina and brain. Prog Retin Eye Res. 2019;70:1‐22. 30999027 10.1016/j.preteyeres.2019.04.001

[43] Vosgha H , Ariana A , Smith RA , Lam AK . miR‐205 targets angiogenesis and EMT concurrently in anaplastic thyroid carcinoma. Endocr Relat Cancer. 2018;25:323‐337. 29317480 10.1530/ERC-17-0497

[44] Zhang P , Lu X , Shi Z , et al. miR‐205‐5p regulates epithelial‐mesenchymal transition by targeting PTEN via PI3K/AKT signaling pathway in cisplatin‐resistant nasopharyngeal carcinoma cells. Gene. 2019;710:103‐113. 31158447 10.1016/j.gene.2019.05.058

[45] Klionsky DJ , Abdelmohsen K , Abe A , et al. Guidelines for the use and interpretation of assays for monitoring autophagy. Autophagy. 2016;12(1):1‐222. 26799652 10.1080/15548627.2015.1100356 PMC4835977

[46] Ferrington DA , Sinha D , Kaarniranta K . Defects in retinal pigment epithelial cell proteolysis and the pathology associated with age‐related macular degeneration. Prog Retin Eye Res. 2016;51:69‐89. 26344735 10.1016/j.preteyeres.2015.09.002 PMC4769684

[47] Hirasawa M , Noda K , Noda S , et al. Transcriptional factors associated with epithelial‐mesenchymal transition in choroidal neovascularization. Mol Vis. 2011;17:1222‐1230. 21617757 PMC3102030

[48] Qu S , Wang T , Huang J . Presence of the minor allele of microRNA205 rs3842530 polymorphism increases 18FDG uptake in patients with breast cancer via targeting VEGF. Mol Med Rep. 2018;17:636‐642. 29115451 10.3892/mmr.2017.7914

[49] Brown JA , Bourke E . Practical bioinformatics analysis of miRNA data using online tools. Methods Mol Biol. 2017;1509:195‐208. 27826929 10.1007/978-1-4939-6524-3_18

[50] Ren L , Wei C , Li K , Lu Z . LncRNA MALAT1 up‐regulates VEGF‐A and ANGPT2 to promote angiogenesis in brain microvascular endothelial cells against oxygen‐glucose deprivation via targetting miR‐145. Biosci Rep. 2019;39:BSR20180226. 30038058 10.1042/BSR20180226 PMC6400790

[51] Ertekin S , Yildirim O , Dinc E , Ayaz L , Fidanci SB , Tamer L . Evaluation of circulating miRNAs in wet age‐related macular degeneration. Mol Vis. 2014;20:1057‐1066. 25221421 PMC4113960

[52] Strafella C , Errichiello V , Caputo V , et al. The interplay between miRNA‐related variants and age‐related macular degeneration: evidence of association of MIR146A and MIR27A. Int J Mol Sci. 2019;20:E1578. 30934838 10.3390/ijms20071578 PMC6480223

[53] Cascella R , Strafella C , Longo G , et al. Uncovering genetic and non‐genetic biomarkers specific for exudative age‐related macular degeneration: significant association of twelve variants. Oncotarget. 2017;9:7812‐7821. 29487693 10.18632/oncotarget.23241 PMC5814260

[54] Cascella R , Strafella C , Caputo V , et al. Towards the application of precision medicine in age‐related macular degeneration. Prog Retin Eye Res. 2018;63:132‐146. 29197628 10.1016/j.preteyeres.2017.11.004

[55] Grassmann F , Schoenberger PG , Brandl C , et al. A circulating microrna profile is associated with late‐stage neovascular age‐related macular degeneration. PLoS ONE. 2014;9:e107461. 25203061 10.1371/journal.pone.0107461 PMC4159338

[56] Menard C , Rezende FA , Miloudi K , et al. MicroRNA signatures in vitreous humour and plasma of patients with exudative AMD. Oncotarget. 2016;7:19171‐19184. 27015561 10.18632/oncotarget.8280 PMC4991373

[57] Eandi CM , Alovisi C , De Sanctis U , Grignolo FM . Treatment for neovascular age related macular degeneration: the state of the art. Eur J Pharmacol. 2016;787:78‐83. 26948315 10.1016/j.ejphar.2016.03.002

[58] Papudesu C , Clemons TE , Agron E , Chew EY . Association of mortality with ocular diseases and visual impairment in the age‐related eye disease study 2: age‐related eye disease study 2 report number 13. Ophthalmology. 2018;125:512‐521. 29153456 10.1016/j.ophtha.2017.10.028 PMC5866182

[59] Dalvin LA , Starr MR , AbouChehade JE , et al. Association of intravitreal anti‐vascular endothelial growth factor therapy with risk of stroke, myocardial infarction, and death in patients with exudative age‐related macular degeneration. JAMA Ophthalmol. 2019;137:483. 30703203 10.1001/jamaophthalmol.2018.6891 PMC6512306

[60] Gopinath B , Liew G , Burlutsky G , Mitchell P . Age‐related macular degeneration and risk of total and cause‐specific mortality over 15 years. Maturitas. 2016;84:63‐67. 26596903 10.1016/j.maturitas.2015.11.001

[61] Solomon SD , Lindsley KB , Krzystolik MG , Vedula SS , Hawkins BS . Intravitreal bevacizumab versus ranibizumab for treatment of neovascular age‐related macular degeneration: findings from a cochrane systematic review. Ophthalmology. 2016;123(70–77):e71. 26477843 10.1016/j.ophtha.2015.09.002 PMC4695272

[62] McGuinness MB , Finger RP , Karahalios A , et al. Age‐related macular degeneration and mortality: the Melbourne Collaborative Cohort Study. Eye. 2017;31:1345‐1357. 28820184 10.1038/eye.2017.139 PMC5601439

[63] Ferris FL , Wilkinson CP , Bird A , et al. Beckman initiative for macular research classification committee. Clinical classification of age‐related macular degeneration. Ophthalmology. 2013;120:844‐851. 23332590 10.1016/j.ophtha.2012.10.036 PMC11551519

