# Supplementary material for: Expression of VEGFA‐regulating miRNAs and mortality in wet AMD
Source: J Cell Mol Med. 2019 Oct 21;23(12):8464–71. doi: 10.1111/jcmm.14731 (PMC6850949; doi:10.1111/jcmm.14731)
Supplement: Supplementary file 1 [file JCMM-23-8464-s001.docx]

**Supplement Table 1.** Baseline characteristics of cataract and cataract-wetAMD surgery patients

|  | Cataract | Cataract + wAMD (anti-VEGF) |
| --- | --- | --- |
| **Total** | **15364** | **330** |
| Age (y) | 77.7±6.3 | 81.9±5.8 |
| Gender (M:F) % | 34:66 | 29:71 |
| Mean follow-up (d) | 1598±1002 | 1360±892 |
| No. of anti-VEGF injections | - | 9.8±8.9 |

Data are given as mean±SD or proportion (%). VEGF, vascular endothelial growth factor; wAMD, wet age-related macular degeneration.

| **Supplement Table 2.** Cox regression analysis | | | | | | | |
| --- | --- | --- | --- | --- | --- | --- | --- |
|  |  |  | Multivariate | | | |  |
| Risk factor |  |  | HR | 95% CI | | *p* |  |
| Age | |  |  | |  |  |  |
| years | |  | 1.07 | | 1.07 – 1.08 | **< 0.001** |  |
| Gender | |  |  | |  |  |  |
| Female | |  | Ref | |  |  |  |
| Male | |  | 1.17 | | 1.07 – 1.28 | **0.001** |  |
| wetAMD | |  |  | |  |  |  |
| No | |  | Ref | |  |  |  |
| Yes | |  | 2.05 | | 1.59 – 2.64 | **< 0.001** |  |

Cox regression analysis. *p* values ≤ 0.05 were considered significant (in bold). HR, hazard ratio; Ref, reference category.
